# The Design of Cement Mortar with Low Capillary Suction: Understanding the Effect of Fine Aggregate and Sodium Silicate

**DOI:** 10.3390/ma15041517

**Published:** 2022-02-17

**Authors:** Natalia Szemiot, Łukasz Sadowski

**Affiliations:** Department of Materials Engineering and Construction Processes, Faculty of Civil Engineering, Wroclaw University of Science and Technology, 50-370 Wroclaw, Poland; nataliaszemiot@gmail.com

**Keywords:** fine aggregate, cement, mortar, capillary suction

## Abstract

The article presents the results of research that was carried out in order to analyze the capillary suction of cement mortar. Capillary suction is a common process that occurs when porous material is in free contact with moisture. The result of high capillary suction may be excessive moisture in buildings, and it is therefore important to limit the causes of such moisture. The main aim of the presented research is to show the influence of sodium silicate (in various proportions), as well as the quantity of aggregate, on capillary suction. Three different types of cement mortar and one type of fine aggregates were analyzed in the research. At the beginning, the capillary suction of the aggregates was analyzed. Afterwards, nine cement mortar bars were made, which were then used to examine the capillary suction. As a result of this study, it was proved that M15 cement mortar with basalt fine aggregate and a higher proportion of sodium silicate was the mortar with the lowest capillary suction. It was found that M15 cement mortar with basalt fine aggregate and a higher proportion of sodium silicate had 39 mm of capillary suction after 120 h of being immersed in water. M5 cement mortar without sodium silicate had the highest index of capillary suction, which shows that adding sodium silicate to cement mortar can significantly reduce capillary suction.

## 1. Introduction

The degradation of buildings caused by excessive moisture is a common problem in the construction industry [1]. Capillary suction is a common process that occurs when porous material is in free contact with moisture. The problem of excessive moisture in walls mostly occurs in old buildings. This is a worldwide problem that is of particular importance in relation to sites that have a high historical value, are legally protected, and have cultural heritage. The most important effect of capillary suction in buildings is the appearance of moisture in the lower part of the buildings’ walls. Moisture in the structure of the wall, as a result of capillary suction, penetrates into the dry parts of the wall, and causes damage as a result [2,3]. Capillary suction can even cause building failure [4,5,6].

**Figure 1:**
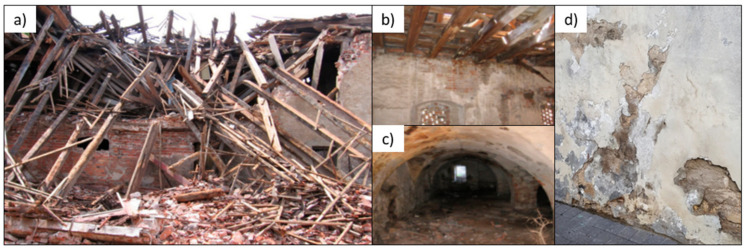
Examples of moisture on buildings: (**a**) granary building—basement [2]; (**b**) granary building—1st floor [2]; (**c**) a building after disaster [2]; (**d**) damage of bricks and mortar caused by excessive moisture [1].

Figure 1a,d presents the possible consequences of moisture in buildings.

Every type of cement has a different mineral structure, which should be taken into account at the stage of formulating design assumptions with regard to the place in which the concrete mixture will be used. This is due to the fact that each type of cement has various characteristic features, such as a different resistance to aggressive environments, a different heat of hydration, and the possibility of being used in a composite with various admixtures or additives. The choice of the type of cement also depends on the microstructural image of the hardened mortars. The most commonly used types of cement mortars are M5, M10 and M15 mortars.

In the presented study, CEM I Portland cement was used to make the masonry mortar [7]. CEM I Portland cement was obtained by grinding Portland clinker with the addition of about 5% of calcium sulphate dihydrate or anhydrite, and is a bond commonly used in construction (approx. 40%) [8]. CEM I Portland cement 42.5 R, which was used to conduct the research, is characterized by a rapid increase in durability, a high heat of hydration and a short setting time. It is used in the production of concretes of classes B20-B50, the execution of monolithic structures, and the production of prefabricated elements [8,9,10].

There are methods of lowering the capillary suction index that are known from the literature. Various types of additives and admixtures added to the cement mortar are used, e.g., polypropylene fibers. However, there has been no research on the reduction in the capillary suction index of cement mortar with the use of sodium silicate. The main benefit of using sodium silicate is that it enables a significant reduction in the capillary suction index of the mortar. Sodium silicate is added to the cement mortar in very small amounts, due to which the costs associated with its use are low. It is a non-toxic, non-hazardous and environmentally friendly product. In addition, sodium silicate does not react with other compounds, which means it is relatively stable. Table 1 presents selected articles concerning capillary suction.

The authors of the articles listed in Table 1 used, among others, powdered silicone and sodium oleate for their research regarding capillary suction. Sodium silicate was not used in any of the above-mentioned research to protect against capillary suction. Different types of admixtures and cement mortars were used in these studies. The research was also carried out with the use of recycled materials. However, none of the mentioned authors used sodium silicate. Capillary suction is common in old buildings, and its visible effect is moisture in the lower parts of a building’s walls. Solutions that can be found in the literature show how the capillary suction index can be lowered.

However, the solutions presented in the above table are more expensive and complicated than the one proposed by the authors of this article. Therefore, the novelty of this study involves the designing of a cement mortar with the addition of sodium silicate in order to reduce the capillary suction index. Moreover, the goal of the research was to demonstrate the impact of the use of different proportions of sodium silicate and basalt fine aggregate on capillary suction. The main aim of the presented research is to show the influence of sodium silicate (in various proportions) and the quantity of aggregate on capillary suction. What is the impact of sodium silicate on the capillary suction of cement mortar? Does the type of cement mortar (amount of fine aggregate used) affect the height of the capillary suction?

Considering the above, the main aim of this research is to analyze the effect of selected properties of mortar components on the capillary suction of cement masonry mortar. The aim is also to find out the effect of sodium silicate admixture on the capillary suction of cement mortar.

## 2. Materials and Methods

### 2.1. Basalt Fine Aggregate

**Figure 2:**
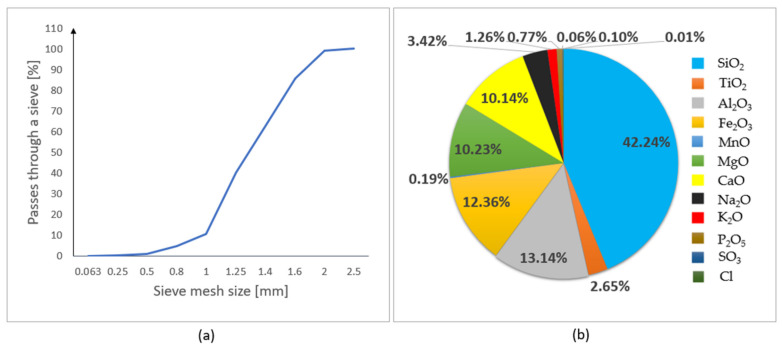
Properties of the basalt fine aggregate: (**a**) particle size distribution; (**b**) chemical composition.

In this research, basalt fine aggregate with a density of ρ_d_ = 3.07 g/cm^3^ was used. Basalt fine aggregate is produced from effusive igneous rock (based on the information provided in [28]). Figure 2 shows the particle size and chemical composition of the basalt fine aggregate. The grain size of the basalt fine aggregate varies between 0.25 and 2.5 mm. The dominant element in the composition of basalt fine aggregate is SiO_2_ (42.24%).

### 2.2. Cement

In this research, Portland cement CEM I 42.5 R was used. The chemical and physical properties of the cement are shown in Table 2.

**Figure 3:**
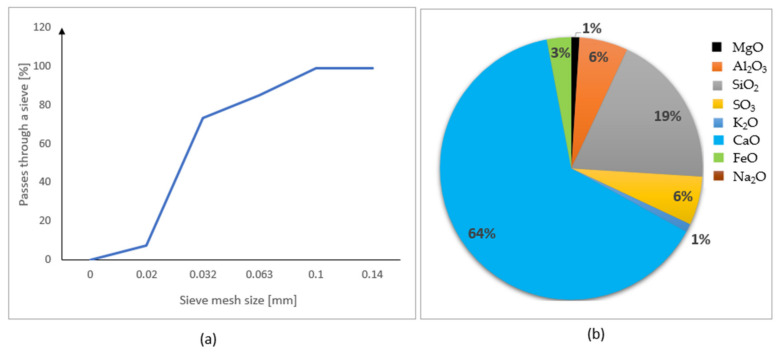
Properties of the Cement CEM I 42.5 R: (**a**) particle size distribution; (**b**) chemical composition (based on the data provided in [30]).

Figure 3a,b shows the particle size distribution and chemical composition of the Cement CEM I 42.5 R. The grain size of cement varies between 0.02 and 0.14 mm. The dominant element in the composition of cement is CaO (64%).

### 2.3. Sodium Silicate

In this research, Dragon R-145 sodium silicate (Na_2_O + SiO_2_) was used. According to the product’s label, it is also called silicic acid, silicic salt, or sodium silicate solution. As stated by the producer, it is an isolator against water absorption. Sodium silicate protects cement mortar against moisture. Table 3 shows the chemical and physical properties of the sodium silicate.

### 2.4. Determination of the Capillary Suction of the Fine Aggregate

For the capillary suction test, a 1.5 m long pipe made of plexiglass with an internal diameter of 143 mm was used. The lower end of the pipe was finished with geotextile. The pipe was filled with fine aggregate up to the height of 1 m. Then, having previously prepared a large vessel filled with water up to 1 cm, the pipe with the fine aggregate was installed in the vessel.

For the capillary suction test, basalt fine aggregate was used, which had a density volume (ρ_d_) of 3.07 g/cm^3^.

**Figure 4:**
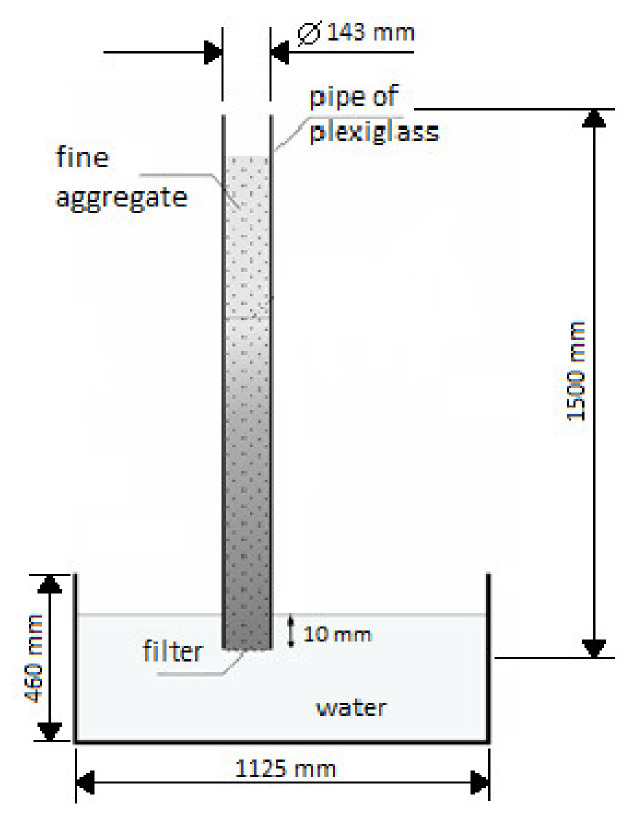
Diagram of the experimental test of the fine aggregate (own elaboration based on [32]).

Figure 4 shows a diagram of the experimental test stand for testing the capillary suction of the fine aggregate.

The capillary suction test was carried out on basalt fine aggregate, from which 3 different samples were taken in order to conduct the capillary suction test.

### 2.5. Preparation of Cement Mortar Bars

According to PN-EN 480-1, 40 mm × 40 mm × 160 mm bars were prepared.

Table 4 shows the proportions of the M5, M10, and M15 masonry mortars that were used to make the bars.

First, a cement masonry mix was made, which was then transferred to appropriate forms (bar dimensions 40 mm × 40 mm× 160 mm). Then, 24 h later, the bars were disassembled and moved in order to mature for 28 days. After 28 days of maturation, a capillary suction test was conducted.

### 2.6. Determination of the Capillary Suction of the Cement Masonry Mortar

The bars (40 mm × 40 mm × 160 mm) made from the cement masonry mortar were transferred to a bath with water and immersed up to the height of 1 cm. After 5 min, 15 min, 30 min, 1 h, 2 h, 3 h, 5 h, 10 h, 24 h, 48 h, 72 h, 96 h and 120 h, respectively, the height of the capillary suction was measured.

**Figure 5:**
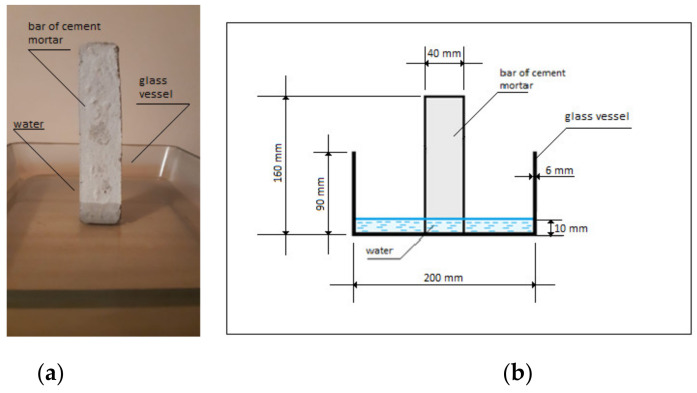
A cement masonry mortar bar: (**a**) side view; (**b**) scheme of the stand for the capillary suction test of the cement masonry mortar.

Figure 5a shows the scheme of the stand for the capillary suction test of the cement masonry mortar bars (the side view). Figure 5b shows the scheme of the stand for the capillary suction test of the cement masonry mortar.

## 3. Results

In order to design a cement mortar with a low capillary suction, the capillary suction of the fine aggregate should first be analyzed. This is due to the fact that fine aggregate is a component of cement mortar.

### 3.1. The Capillary Suction of the Basalt Fine Aggregate

Table 5 shows the results of the research on the increase in capillary suction, which was conducted on basalt fine aggregate (three samples of basalt fine aggregate were taken). The capillary suction was measured in a specific time and given in millimeters. The last column shows the average capillary suction from the three samples. At the early stage of the research, each sample showed a rapid growth of capillary suction, but this growth slowed down after time.

**Figure 6:**
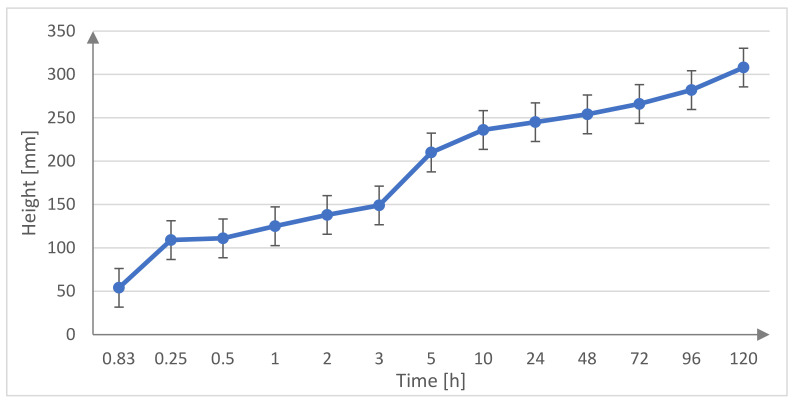
Capillary suction of the basalt fine aggregate in relation to time.

The line graph of the capillary suction shown below (Figure 6) presents the level of increase in water with regard to a specific time. In the first minutes, the values of the capillary suction index increase rapidly, with time the index of capillary suction increases slowly.

### 3.2. The Capillary Suction of the Cement Masonry Mortars

**Figure 7:**
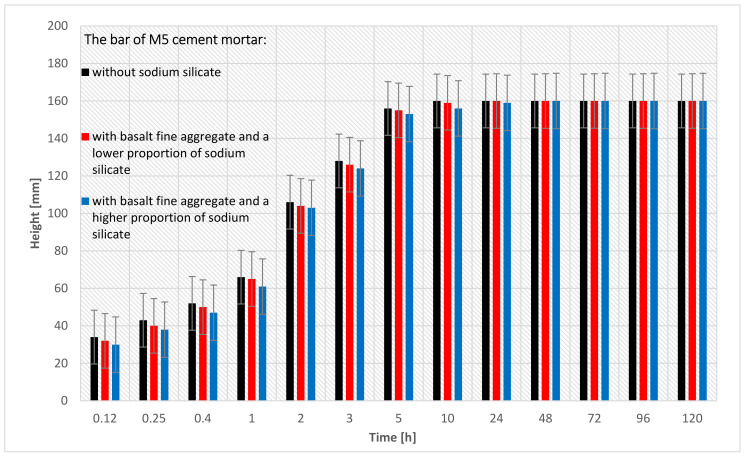
The results of the capillary suction of the M5 cement mortar bar with basalt fine aggregate: without sodium silicate, with a lower proportion of sodium silicate, and with a higher proportion of sodium silicate.

Figure 7 shows the capillary suction of the M5 cement mortar in the form of a bar graph. Three pieces of M5 cement masonry mortar bars were tested (for the precise specification of the bars, see Table 5). In this research, each of the three bars was measured 13 times. The cement mortar bar with the basalt fine aggregate became soaked after 10 h.

**Figure 8:**
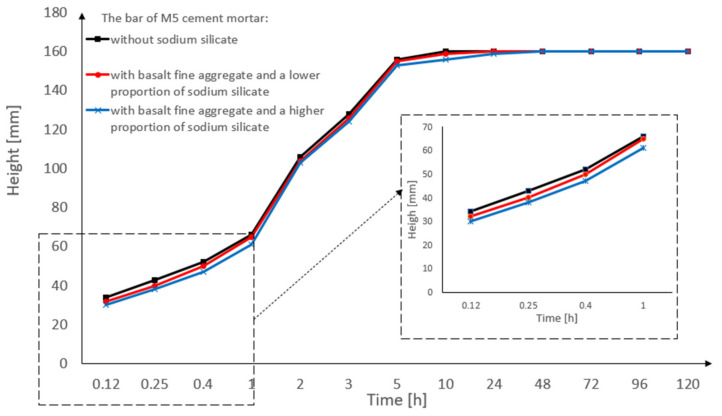
Capillary suction of the M5 cement mortar bar with the basalt fine aggregate: without sodium silicate, with a lower proportion of sodium silicate, and with a higher proportion of sodium silicate.

Figure 8 shows the capillary suction of the M5 cement mortar in the form of a bar graph. Basalt fine aggregate and sodium silicate in various proportions were used (for the precise specification of the bars-see Table 5). Each type of bar obtained very similar results, irrespective of the amount of sodium silicate. A graph of the capillary suction in the first hour of the research is also shown. The results of the bars are very similar to each other. In the M5 cement mortar bar, the impact of the sodium silicate on capillary suction is not visible. The reason for these results is the amount of fine aggregate used in the cement mortar bars.

**Figure 9:**
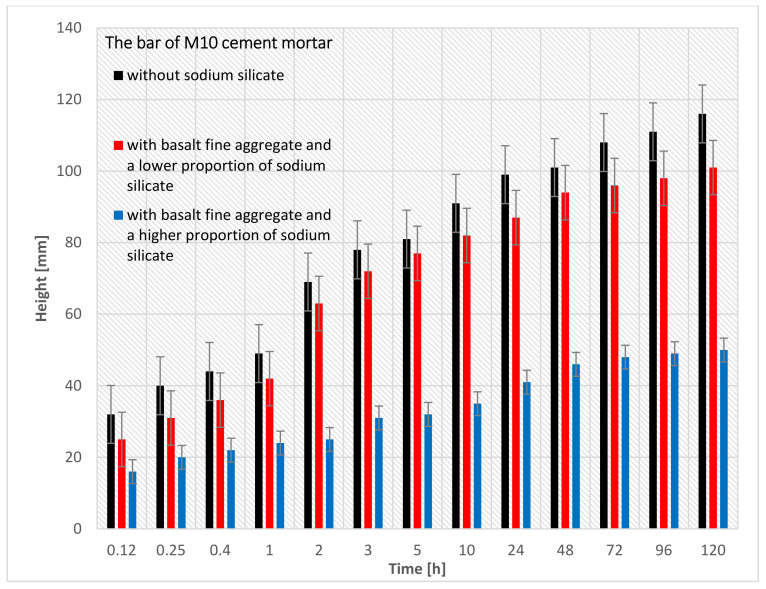
The results of the capillary suction of the M10 cement mortar bar with basalt fine aggregate: without sodium silicate, with a lower proportion of sodium silicate, and with a higher proportion of sodium silicate.

Figure 9 shows the results of the capillary suction study, in which three pieces of M10 cement masonry mortar bars were used (for the precise specification of the bars, see Table 5). In this research, each of the three bars was measured 13 times. The numbers below the graph represent the respective bars that were described Table 5.

By analyzing Figure 9, it can be seen that bar nr 19 (M10 cement mortar with basalt fine aggregate and a higher proportion of sodium silicate) had the lowest capillary suction (50 mm) after 120 h of being immersed in the water. On the other hand, bar nr 13 (M10 cement mortar with basalt fine aggregate without sodium silicate) had the highest capillary suction (116 mm) after 120 h of being immersed. The reason for the lower capillary suction of the bar nr 19 is that more sodium silicate can be added to the cement mortar.

**Figure 10:**
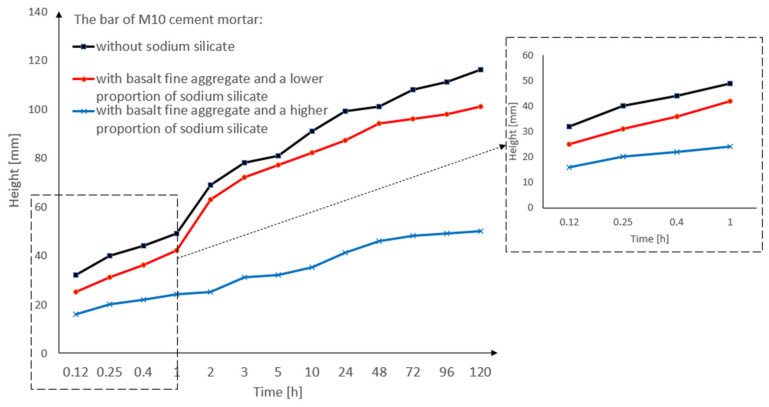
Capillary suction of the M10 cement mortar bar with the basalt fine aggregate: without sodium silicate, with a lower proportion of sodium silicate, with a higher proportion of sodium silicate.

Figure 10 shows the capillary suction of the M10 cement mortar in the form of a bar graph. Basalt fine aggregate and sodium silicate were used in various proportions (for the precise specification of the bars, see Table 5). The cement mortar bar with the basalt fine aggregate (without sodium silicate), and the bar with the basalt fine aggregate with a greater proportion of sodium silicate, had similar results. However, the bar with basalt fine aggregate and a greater proportion of sodium silicate had a lower capillary suction than the other bars. The cement mortar bars without sodium silicate and with a lower proportion of sodium silicate had the highest levels of capillary suction, and their values, as shown in the graph, definitely differ from the cement mortar bar with a higher proportion of sodium silicate.

**Figure 11:**
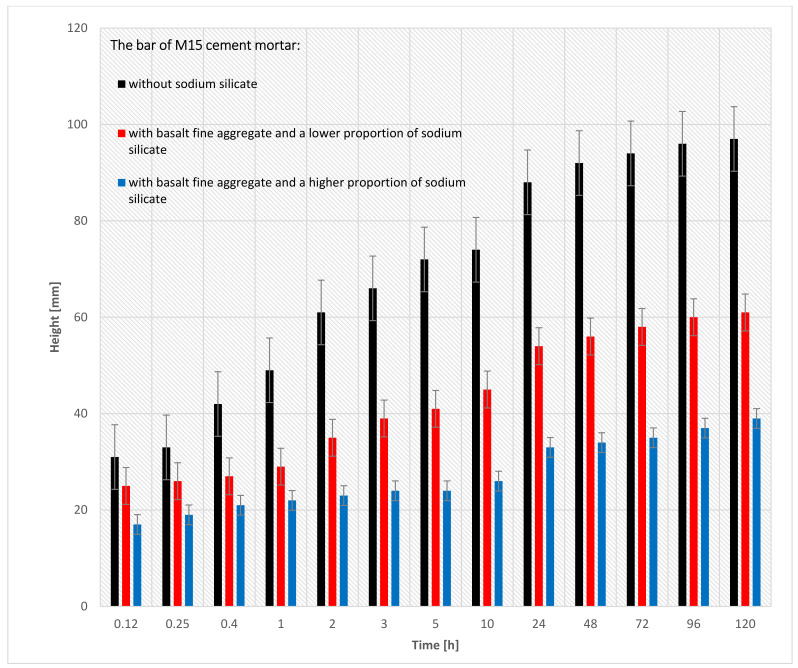
The results of the capillary suction of the M15 cement mortar bar with basalt fine aggregate: without sodium silicate, with a lower proportion of sodium silicate, with a higher proportion of sodium silicate.

Figure 11 shows the results of the capillary suction study in which three M15 cement masonry mortar bars were used (for the precise specification of the bars, see Table 5). In this research, each of the three bars was measured 13 times.

The capillary suction of the M15 cement masonry mortar is shown in the bar chart in Figure 11. Table 4 describes the characteristics of a given bar class (the mixture of concrete components affects the capillary rise results), while Table 5 describes the test results that show the time in which a given bar of concrete mortar reached a specific level of capillary rise.

When analyzing Figure 11, bar nr 29 (M15 cement mortar with basalt fine aggregate and a higher proportion of sodium silicate) had the lowest capillary suction (39 mm) after 120 h of being immersed in the water. On the other hand, bar nr 23 (M15 cement mortar with basalt fine aggregate without sodium silicate) had the highest capillary suction (97 mm) after 120 h of being immersed. The reason for the lower capillary suction of the bar nr 19 is that more sodium silicate can be added to the cement mortar.

**Figure 12:**
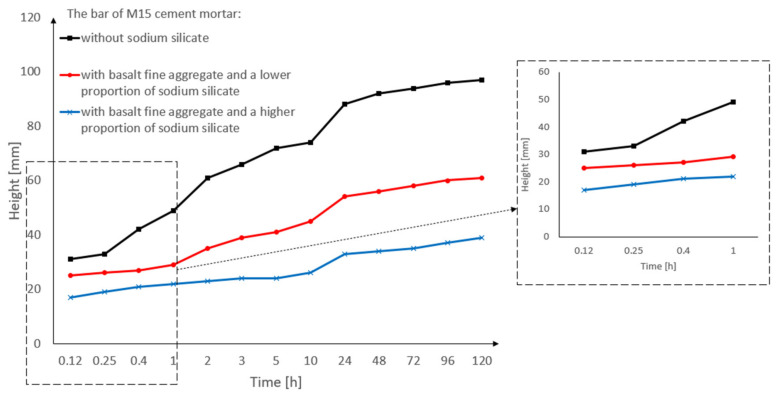
Capillary suction of the M15 cement mortar bar with basalt fine aggregate: without sodium silicate, with a lower proportion of sodium silicate, and with a higher proportion of sodium silicate.

Figure 12 shows the capillary suction of the M15 cement mortar in the form of a bar graph. The basalt fine aggregate and sodium silicate were used in various proportions (for the precise specification of the bars, see Table 5). The cement mortar bar with the basalt fine aggregate (without sodium silicate) had the highest capillary suction when compared to the other bars. However, the bar with basalt fine aggregate and a greater proportion of sodium silicate had a lower capillary suction than the other bars. The cement mortar bar without the sodium silicate had the highest level of capillary suction, and its values definitely differ from the cement mortar bar with the sodium silicate. Tests of the capillary suction showed that the M15 mortar, when compared to the M5 and M10 mortars, had the lowest rate of capillary suction. The reason for this was the proportions of the components that were used in the mortar when compared to the M5 and M10 mortars.

**Figure 13:**
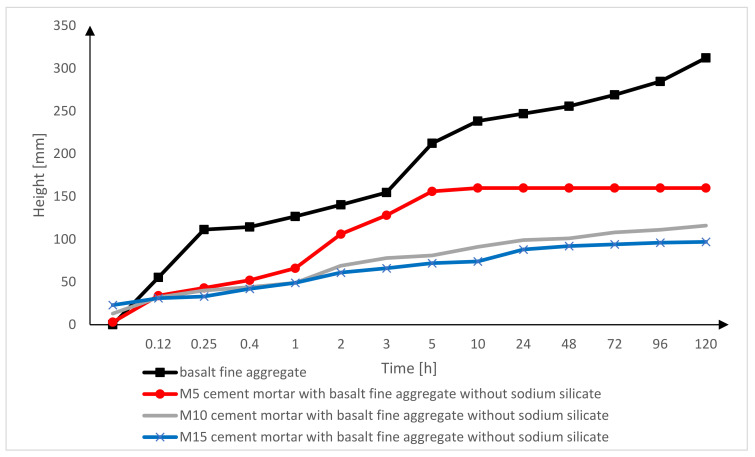
Comparison of the results of the capillary suction of the M5, M10, M15 cement mortar bars without sodium silicate with basalt fine aggregate.

Figure 13 shows the results of the capillary suction of only the basalt fine aggregate, as well as the results of the capillary suction of the M5, M10 and M15 cement masonry mortars without sodium silicate with the basalt fine aggregate.

The fine aggregate had the highest level of capillary suction, and its values definitely differ from the cement mortar bars. This situation can be caused by the components of the cement mortars. In order to prepare the cement mortar bars, apart from fine aggregate, cement mortar was used. Therefore, in the bars made of cement mortar, the cement and water were used. For this reason, the capillary suction level is less in the aggregates. The finer the aggregates in the cement mortar, the higher the capillary suction index.

**Figure 14:**
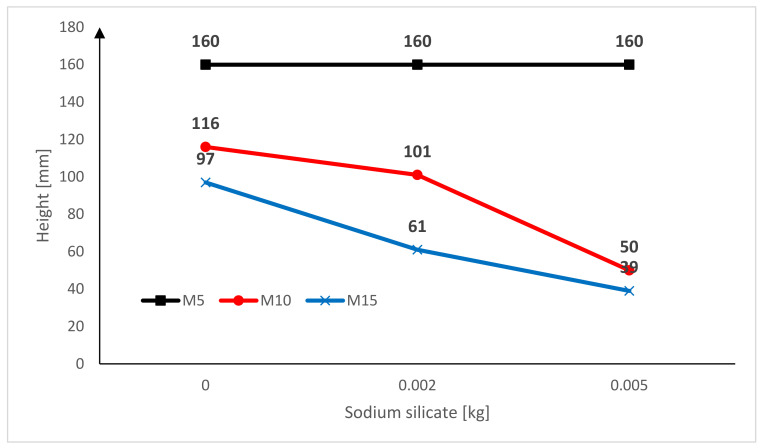
The effect of the sodium silicate on the capillary suction index of the M5, M10, M15 cement mortar bars after 120 h of being immersed.

Figure 14 shows the effect of the sodium silicate on the capillary suction index of the M5, M10, M15 cement mortar bars after 120 h of being immersed. The graph shows that the type of used cement mortar has an effect on the capillary suction index. After 120 h of testing, the M5 mortar achieved the highest index of capillary suction (without sodium silicate, with a lower proportion of sodium silicate, and with a higher proportion of sodium silicate). In the M5 cement mortar bar, the impact of the sodium silicate on capillary suction is not visible. The impact of the sodium silicate can be seen in the M10 and M15 mortars. M15 cement mortar bar with basalt fine aggregate and a higher proportion of sodium silicate had the lowest capillary suction (39 mm) after 120 h of being immersed in the water. Adding sodium silicate to the cement mortar bar had a positive effect on the capillary suction index.

## 4. Conclusions

The main aim of the presented research is to show the influence of sodium silicate (in various proportions), as well as the quantity of aggregate, on capillary suction. For the purpose of this article, three different types of cement mortars (M5, M10 and M15 cement mortars) and one type of fine-grained aggregate (basalt fine aggregate) were analyzed.

- As a result of the research, it was found that the M15 cement mortar with the fine basalt aggregate and more sodium silicate had the lowest rate of capillary suction. The reason for this was the sodium silicate added to the cement mortar. The amount of aggregate added to the cement mortar is also important. When compared to the M5 and M10 mortars, the M15 mortar contains the smallest amount of fine aggregate.
- Tests of the capillary suction showed that the M5 mortar, in comparison to the M10 and M15 mortars, had the highest rate of capillary suction. The reason for this was the proportions of the components that were used in the mortar when compared to the M10 and M15 mortars.
- In the case of the M10 mortar, the best result was obtained by the bar made of the cement mortar with basalt fine aggregate and more sodium silicate, while in the case of the M15 mortar, the best result was obtained by the bar with basalt fine aggregate and more sodium silicate. The reason for this was the sodium silicate added to the cement mortar.
- An important element that enabled a favorable result to be obtained was sodium silicate. This, as shown by the research, made it possible to reduce water absorption.

To sum up, the most advantageous mortar for making cement composites with a low capillary suction is the M15 mortar. This is due to the fact that it has the smallest amount of fine aggregate (which affects water rising) when compared to the M5 and M10 mortars. An important element in the design of this mortar is also the admixture of sodium silicate, which, as the research shows, has a significant impact on the rise of water. Ultimately, in the presented studies, one bar obtained the best result: the M15 cement mortar bar with the basalt fine aggregate and more sodium silicate.

However, more research should be conducted with regard to other fine aggregates, e.g., granite fine aggregates [30]. Tests concerning the method of propagation of the injection mass with regard to the used cement mortar and brick (the brick’s microstructure) should also be considered.

## Figures and Tables

**Table 1:** Current knowledge on capillary suction in cement mortars (based on [11,12,13,14,15,16,17,18,19,20,21,22,23,24,25,26,27]).

No.	Author	Main Findings
1	Knarud et al. [11]	When tested with their face down, it was found that pillar specimens (consisting of three bricks with mortar joints) had higher water absorption coefficients than individual bricks for all test series.
2	Guimarãesa et al. [12]	The results of the experimental campaign of absorption in samples of clay brick with and without joints, and also clay brick with joints with different contact configurations showed that when moisture reaches the interface there is a slowing of the wetting process due to the hygric resistance of the interface.
3	Castro Mendes et al. [13]	Macropores are advantageous in the case of lightweight mortars with smaller thermal conductivity, water absorption and capillarity. The decrease in the inlet of water leads to a smaller chance of moisture problems.
4	Lanzón et al. [14]	Powdered silicone and sodium oleate showed the best resistance to water penetration, while metallic soaps in the form of calcium stearate and zinc stearate showed the lowest efficiency at low dosages.
5	Veiga et al. [15]	The old lime mortars studied in the research had capillarity coefficients that are similar to new lime mortars (between 90 and 10 min). However, they had much slower absorptions during the first few minutes. This means that the absorption rate of old mortars remains almost constant during the first 90 min, while new mortars have a much higher rate of absorption during the first few minutes, after which they stabilize.
6	Xiao et al. [16]	The results showed that when the freeze–thaw environment is the same, the replacement rate of the recycled coarse aggregate is faster, the cumulative water absorption by the RAC is greater, and the initial water absorption (capillary rise) is faster. When the freeze–thaw environment is different, there are more freeze–thaw cycles, the accumulated water absorption (capillary rise) by the RAC at the same replacement rate of recycled coarse aggregate is greater, and the initial water absorption (capillary rise) is faster.
7	Rirsch et al. [17]	It was found that the characteristics of mortar considerably affect the height of rising damp. Additionally, a strong correlation between rising damp and the Sharp Front Model was observed. It was also found that the rate of absorption of water into the mortar is a crucial factor in determining the height of the rising damp.
8	Belleghem et al. [18]	The entrance of capillary water into the mortar (cracked and uncracked) was simulated using the 3D Richards equation together with the finite element method (FEM). The developed model was characterized by realistic boundary conditions of the process of water evaporation. The model was validated using gravimetrical water absorption and X-ray radiography.
9	Morón et al. [19]	According to the results of the studies, recycled mortars show a higher water absorption during capillary action due to the larger capillary system formed in this type of mortar (as a result of the high absorption of recycled aggregates).
10	Santamaría-Vicario et al. [20]	The results of the study indicate that the joint use of both steelmaking slags and additives enables the production of masonry mortars with low water absorption by capillarity index, with water vapor permeability being high. Mortars made with EAFS and LFS slags need additives in order to retain mixing water and to ensure their correct setting and hardening.

**Table 2:** The chemical and physical properties of the Portland cement CEM I 42.5 R (based on the information provided in [29]).

Name of Property	Unit	Average Value	Requirement
Setting time	(min)	251	≥60
Consistency	(%)	27.4	No requirements
Specific surface area	(cm^2^/g)	3655	No requirements
Content of SO_3_	(%)	2.76	≤4.0%
Content of Cl^−^	(%)	0.045	≤0.10%
Content of Na_2_O_eq_	(%)	0.50	No requirements

**Table 3:** The chemical and physical properties of the sodium silicate (own study based on the data provided in [31]).

Appearance:	White, colorless or semi-translucent liquid
Odor:	Odorless
pH:	11–13 in a temperature of 20 °C
Melting point/pour point [°C]:	For a pure substance: Softening point 550–670 °C Pour point 730–870 °C
Flash point:	Non-flammable substance
Upper/lower flammability/explosive limits:	Research is not necessary—non-flammable substance
Explosive properties:	Research is not necessary—inorganic substance
Breakdown temperature:	No data—the substance does not decompose at temperatures below 1400 °C
Solubility:	Sodium silicate water solution—sodium silicate is mixed with water in any ratio.

**Table 4:** Mix designs of the masonry mortars.

No.	Cement (kg)	Water (kg)	Fine Aggregate (kg)	Sodium Silicate (kg)
**M5 cement mortar**				
3	0.5	0.48	3.5	0
6	0.5	0.48	3.5	0.002
9	0.5	0.48	3.5	0.005
**M10 cement mortar**				
13	0.5	0.44	2.86	0
16	0.5	0.44	2.86	0.002
19	0.5	0.44	2.86	0.005
**M15 cement mortar**				
23	0.5	0.34	2.14	0
26	0.5	0.34	2.14	0.002
29	0.5	0.34	2.14	0.005

**Table 5:** The results of the capillary suction of the basalt fine aggregate.

Basalt Fine Aggregate				
	h (mm)			
Time	Sample 1	Sample 2	Sample 3	Average
5 min.	54	56	56	55.33
15 min.	109	113	112	111.33
30 min.	111	116	116	114.33
1 h	125	128	127	126.67
2 h	138	141	142	140.33
3 h	149	156	159	154.67
5 h	210	214	213	212.33
10 h	236	240	239	238.33
24 h	245	249	247	247.00
48 h	254	258	255	255.67
72 h	266	270	271	269.00
96 h	282	286	286	284.67
120 h	308	315	314	312.33

## Data Availability

Not applicable.
